# Racial Impact of Hypertension on Colorectal Cancer Screening in Central Illinois, United States

**DOI:** 10.7759/cureus.82190

**Published:** 2025-04-13

**Authors:** Oladoyin Ogunbayo Jolaoye, Sonu Dhillon

**Affiliations:** 1 Internal Medicine - Pediatrics, University of Illinois College of Medicine at Peoria - Order of St. Francis (OSF) Saint Francis Medical Center, Peoria, USA; 2 Gastroenterology, University of Illinois College of Medicine at Peoria - Order of St. Francis (OSF) Saint Francis Medical Center, Peoria, USA

**Keywords:** colorectal cancer, crc, hypertension, preventative, race, screening

## Abstract

Introduction

Colorectal cancer (CRC) is one of the most common types of cancer in the United States. We evaluated the association between hypertension and CRC screening in the American population in Central Illinois. We performed the analysis by investigating the association between hypertension and CRC screening in the American population in Central Illinois, specifically African Americans, to detect disparities.

Methods

Using electronic medical records from an Illinois healthcare system, we analyzed patients aged 45-75 years between January 2014 and December 2023. The data reviewed included factors such as race, age, gender, education, and hypertension. Race categories were White, African American, and others including Hispanics, Asians, Native Americans, Pacific Islanders, Samoans, and unclassified races. Exclusions were patients outside the age limit or those diagnosed with CRC without screening. We recorded frequencies and percentages for categorical variables and statistical measures for numeric ones. The Pearson chi-squared test evaluated the association between race and CRC screening. Data analysis was performed in R at a 5% significance level.

Results

Among screened patients, 2,264 (75.4%) African American, 14,446 (52.1%) White, and 650 (48.4%) others had hypertension. Of those testing positive for CRC, 14 (73*.*7%) African American, 1 (25%) "others," and 53 (52%) White patients had hypertension. Overall, 68 (54.4%) of the CRC-positive population had hypertension. An association between hypertension and CRC screening was found (p < 0.001), with higher screening rates among hypertensive African Americans compared to other races. Though hypertensive African Americans had higher CRC positivity rates, the small case numbers warrant validation in larger cohorts.

Conclusion

In Central Illinois, African Americans showed the highest rates of hypertension and positive CRC diagnoses in the screened population. They are at higher risk of CRC when hypertensive. Targeted community-based screening programs for hypertensive African Americans, coupled with culturally tailored education on CRC prevention, may reduce disparities in early detection and outcomes.

## Introduction

In the United States of America, Colorectal Cancer (CRC) is one of the most prevalent types of cancer in adult males and females [1]. CRC ranks as the third most common cancer in the United States and is the second leading cause of cancer-related deaths [2]. The United States Preventive Services Task Force (USPTF) guidelines recommend starting CRC screening at age 45 [3]. Various risk factors have been identified to increase the risk of CRC, including age, sex, family history of CRC, intestinal inflammation, obesity, diabetes, smoking, physical activity, and excessive consumption of alcohol and red meat [4]. The incidence of CRC varies among various races; for instance, African Americans have an increased incidence and mortality rates of CRC compared to other ethnic groups [5,6]. Most CRCs begin as polyps in the colon or rectum, which are mostly benign but can develop into cancer over time [7]. More proximal polyps are observed in African American adults in the United States compared to White adults [8].

Risk factors such as obesity and diabetes may influence blood pressure and cancers, potentially confounding the relationship between hypertension and CRC [9]. Stage 1 hypertension and 2 hypertension have been associated with an elevated risk of CRC [9]. A recent meta-analysis of observational studies indicated that individuals with hypertension had a 15% higher risk of developing CRC [10]. Chronic hypertension may exacerbate CRC risk via vascular endothelial dysfunction, which is a condition where there is damage to the lining of blood vessels that can disrupt the delivery of oxygen and blood flow to the colon [11].

African American adults have a higher prevalence of hypertension and lower blood pressure control compared to White adults [12]. Access to healthcare, quality of care, and health status are not equal in the United States, and these systemic barriers compound disparities in screening uptake among African Americans [12]. Based on these observations, we performed this retrospective analysis study to investigate our hypothesis that hypertension can be used to predict CRC detection disparities in African Americans. We performed the analysis by investigating an association between hypertension and CRC screening in the American population in Central Illinois, specifically African Americans.

## Materials and methods

Study design

We conducted a retrospective analysis using data from the electronic medical records (EMRs) of an academic tertiary healthcare system in Illinois, including information on the gender, age, level of education, and ethnicity of the patients. The Institutional Review Board at the Order of St. Francis (OSF) and the University of Illinois College of Medicine at Peoria reviewed and approved this study (2159714-1).

Study population

We analyzed patients between 45 and 75 years of age from January 2014 to December 2023. The data reviewed included factors such as race, age, gender, education, and hypertension. The races in the EMR data were categorized into White and African American. We grouped Hispanics, Asians, Native Americans, Pacific Islanders, Samoans, and other races not classified into one group. Patients outside the age limit, those screened for other ailments using CRC screening methods but were found to have CRC, and patients diagnosed with CRC who did not use the screening methods were excluded.

Data collection

The EMR data on CRC screening contained information on the gender, age, ethnicity, level of education, and race of the patients. The primary variable was hypertension, associated with race and CRC screening. The data included a metric indicating whether a screened patient of a particular race had hypertension or not. This was further divided into positive or negative test results for CRC screening. The test result was collected for each screened race within the identified race and overall screened population.

Statistical analysis

We recorded the frequency and percentage for all categorical variables and calculated the mean, standard deviation, median, and interquartile range for all numeric variables. The Pearson chi-squared test was used to evaluate the association between race and CRC screening. Data analysis was performed using R software, assuming a two-sided, 5% significance level.

## Results

**Figure 1:**
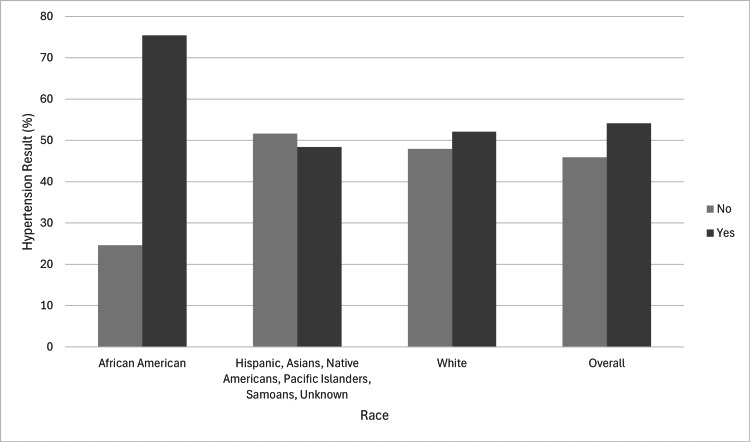
Hypertension characteristics in the screened population compared to patients’ race.

A total of 32,069 patients met the inclusion criteria: 27,726 (86.46%) were White, 3,001 (9.36%) were African American, and 1,342 (4.18%) were categorized as Asian, Native Hawaiian/Pacific (other). In the screened population, 17,147 (53.5%) were females and 14,922 (46.5%) were males, with a median age of 59. Of those screened, 58 (1.9%) of African Americans, 43 (3.2%) of other races, and 340 (1.2%) of White patients had an educational level of less than 12 years. African Americans had the lowest screened population with a Bachelor's degree, with 148 (4.9%), compared to the other races, with 131 (9.8%), and White individuals, with 3,884 (14%). The screening methods used included colonoscopy, Cologuard stool DNA test with fecal immunochemical test (sDNA-FIT), and HC qualitative fecal blood immunoassay (FIT). Of these methods, 2,676 (8.3%) were screened via sDNA-FIT, 22,689 (70.8%) via colonoscopy, and 6,704 (20.9%) via FIT. In the screened population (Table 1), 2,264 (75.4%) of African American patients, 14,446 (52.1%) of White patients, and 650 (48.4%) of races classified as others had hypertension (Figure 1). The number of African American patients without hypertension was 737 (24.6%) compared to 13,280 (47.9%) of White patients and 692 (51.6%) of races classified as other. Overall, 17,360 (54.1%) of the screened population had hypertension, while 14,709 (45.9%) did not have hypertension.

**Table 1:** Summary result of CRC-screened patients in the population *Other races comprised Hispanics, Asians, Native Americans, Pacific Islanders, Samoans, and races not classified CRC, colorectal cancer

Metrics		African American	Other*	White	Overall	P-value
		(N=3,001)	(N=1,342)	(N=27,726)	(N=32,069)	-
Age	Mean (SD)	58.3 (7.63)	57.1 (8.07)	59.5 (8.09)	59.3 (8.07)	
	Median (Q1-Q3)	58.0 (52.0-64.0)	55.0 (51.0-63.0)	59.0 (52.0-66.0)	59.0 (52.0-66.0)	
	Range	[40.0, 75.0]	[40.0, 75.0]	[40.0, 75.0]	[40.0, 75.0]	
Age group (<45)	<45	34 (1.1%)	31 (2.3%)	358 (1.3%)	423 (1.3%)	
	45+	2,967 (98.9%)	1,311 (97.7%)	27,368 (98.7%)	31,646 (98.7%)	
Age group (<50)	<50	240 (8.0%)	175 (13.0%)	1,985 (7.2%)	2,400 (7.5%)	
	50+	2,761 (92.0%)	1,167 (87.0%)	25,741 (92.8%)	29,669 (92.5%)	
Ethnicity	Not Hispanic, Latino/a, or Spanish origin	2,853 (95.1%)	793 (59.1%)	27,403 (98.8%)	31,049 (96.8%)	
	Hispanic, Latino/a, Puerto Rican, or Spanish origin	128 (4.3%)	520 (38.7%)	169 (0.6%)	817 (2.5%)	
	Missing	20 (0.7%)	29 (2.2%)	154 (0.6%)	203 (0.6%)	
Gender	Female	1,680 (56.0%)	731 (54.5%)	14,736 (53.1%)	17,147 (53.5%)	
	Male	1,321 (44.0%)	611 (45.5%)	12,990 (46.9%)	14,922 (46.5%)	
Education	Less than 12 years	58 (1.9%)	43 (3.2%)	340 (1.2%)	441 (1.4%)	
	High school or GED	306 (10.2%)	89 (6.6%)	4,164 (15.0%)	4,559 (14.2%)	
	Some college or associate degree	328 (10.9%)	83 (6.2%)	4,618 (16.7%)	5,029 (15.7%)	
	Bachelor's degree	148 (4.9%)	131 (9.8%)	3,884 (14.0%)	4,163 (13.0%)	
	Graduate degree	102 (3.4%)	172 (12.8%)	2,193 (7.9%)	2,467 (7.7%)	
	Missing	2,059 (68.6%)	824 (61.4%)	12,527 (45.2%)	15,410 (48.1%)	
Hypertension	No	737 (24.6%)	692 (51.6%)	13,280 (47.9%)	14,709 (45.9%)	<0.001
	Yes	2,264 (75.4%)	650 (48.4%)	14,446 (52.1%)	17,360 (54.1%)	

**Figure 2:**
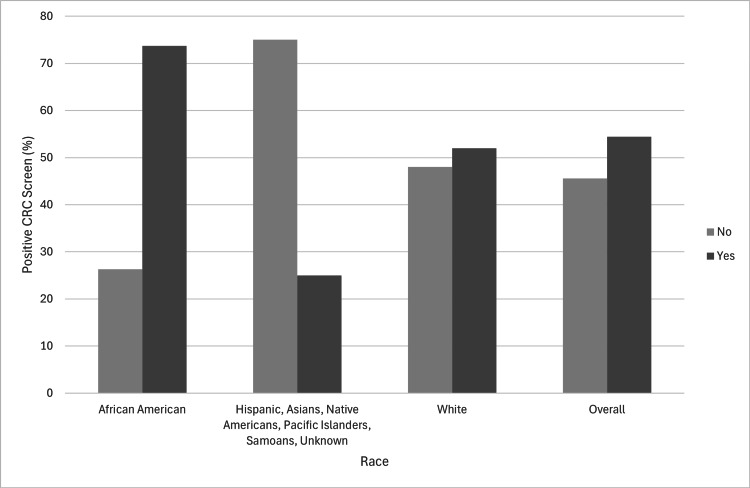
Hypertension characteristics between positive CRC screen results and the different races. CRC, colorectal cancer

The comparison between the races and their hypertension characteristics is shown in Table 2. Among the screened patients who were positive for CRC, 6 (4.8%) had an educational level of less than 12 years, 17 (13.6%) had passed high school or the General Education Development (GED) test, 11 (8.8%) had some college or associate degree, 15 (12.0%) had a Bachelor's degree, and 6 (4.8%) had a graduate degree. Among the screened African American patients who were positive for CRC, 14 (73.7%) had hypertension compared to 5 (26.3%) who did not. In the screened population, 53 (52%) of White patients who tested positive for CRC had hypertension compared to 49 (48%) who did not have hypertension. Overall, 68 (54.4%) screened patients of the total population who tested positive for CRC had hypertension. The data suggested an association between hypertension and CRC screening (p < 0.001). Specifically, African American adults with hypertension had a higher CRC screening rate compared to other races. African American patients with hypertension had the highest percentage of positive CRC tests compared to other races in the data (Figure 2).

**Table 2:** Summary result of participant characteristics by race and screening result *Other races comprised Hispanics, Asians, Native Americans, Pacific Islanders, Samoans, and races not classified

Metrics		African American		Other*		White		Overall	
		Negative	Positive	Negative	Positive	Negative	Positive	Negative	Positive
		(N=2,982)	(N=19)	(N=1,338)	(N=4)	(N=27,624)	(N=102)	(N=31,944)	(N=125)
Age	Mean(SD)	58.3 (7.63)	60.1 (7.33)	57.1 (8.06)	59.5 (10.8)	59.5 (8.09)	60.9 (8.95)	59.3 (8.07)	60.7 (8.71)
	Median (Q1-Q3)	58.0 (52.0-64.0)	61.0 (54.0-67.5)	55.0 (51.0-63.0)	61.0 (56.3-64.3)	59.0 (52.0-66.0)	63.0 (54.0-68.0)	59.0 (52.0-66.0)	62.0 (54.0-68.0)
	Range	[40.0, 75.0]	[47.0, 70.0]	[40.0, 75.0]	[45.0, 71.0]	[40.0, 75.0]	[40.0, 75.0]	[40.0, 75.0]	[40.0, 75.0]
Age group	<45	34 (1.1%)	0 (0%)	31 (2.3%)	0 (0%)	352 (1.3%)	6 (5.9%)	417 (1.3%)	6 (4.8%)
	45+	2,948 (98.9%)	19 (100%)	1,307 (97.7%)	4 (100%)	27,272 (98.7%)	96 (94.1%)	31,527 (98.7%)	119 (95.2%)
Ethnicity	Not Hispanic, Latino/a, or Spanish origin	2,835 (95.1%)	18 (94.7%)	790 (59.0%)	3 (75.0%)	27,301 (98.8%)	102 (100%)	30,926 (96.8%)	123 (98.4%)
	Hispanic, Latino/a, Puerto Rican, or Spanish origin	127 (4.3%)	1 (5.3%)	519 (38.8%)	1 (25.0%)	169 (0.6%)	0 (0%)	815 (2.6%)	2 (1.6%)
	Missing	20 (0.7%)	0 (0%)	29 (2.2%)	0 (0%)	154 (0.6%)	0 (0%)	203 (0.6%)	0 (0%)
Gender	Female	1,667 (55.9%)	13 (68.4%)	729 (54.5%)	2 (50.0%)	14,690 (53.2%)	46 (45.1%)	17,086 (53.5%)	61 (48.8%)
	Male	1,315 (44.1%)	6 (31.6%)	609 (45.5%)	2 (50.0%)	12,934 (46.8%)	56 (54.9%)	14,858 (46.5%)	64 (51.2%)
Education	Less than 12 years	57 (1.9%)	1 (5.3%)	43 (3.2%)	0 (0%)	335 (1.2%)	5 (4.9%)	435 (1.4%)	6 (4.8%)
	High school or GED	306 (10.3%)	0 (0%)	88 (6.6%)	1 (25.0%)	4,148 (15.0%)	16 (15.7%)	4,542 (14.2%)	17 (13.6%)
	Some college or associate degree	328 (11.0%)	0 (0%)	82 (6.1%)	1 (25.0%)	4,608 (16.7%)	10 (9.8%)	5,018 (15.7%)	11 (8.8%)
	Bachelor's degree	148 (5.0%)	0 (0%)	130 (9.7%)	1 (25.0%)	3,870 (14.0%)	14 (13.7%)	4,148 (13.0%)	15 (12.0%)
	Graduate degree	102 (3.4%)	0 (0%)	172 (12.9%)	0 (0%)	2,187 (7.9%)	6 (5.9%)	2,461 (7.7%)	6 (4.8%)
	Missing	2,041 (68.4%)	18 (94.7%)	823 (61.5%)	1 (25.0%)	12,476 (45.2%)	51 (50.0%)	15,340 (48.0%)	70 (56.0%)
Hypertension	No	732 (24.5%)	5 (26.3%)	689 (51.5%)	3 (75.0%)	13,231 (47.9%)	49 (48.0%)	14,652 (45.9%)	57 (45.6%)
	Yes	2,250 (75.5%)	14 (73.7%)	649 (48.5%)	1 (25.0%)	14,393 (52.1%)	53 (52.0%)	17,292 (54.1%)	68 (54.4%)

## Discussion

The data analyzed from a single tertiary healthcare system indicated an association between hypertension and CRC screening. The data demonstrated that African American adults had the highest rate of hypertension and positive CRC screen results. The recommended age for African American adults to get screened for CRC is 45, which is also the recommended age for screening adults with an average risk of CRC [13]. The recommendation to reduce the CRC screening age from 50 to 45 was made by the American College of Gastroenterology (ACG) in 2021; the recommendation applied to individuals at average risk between 45 and 75 years of age [13]. Utilizing the lowered screening age of 45, controlling factors contributing to hypertension, and managing hypertension can effectively decrease the risk of CRC and its mortality [14,15]. The ACG recommended colonoscopy and FIT as the primary CRC screening methods for early detection of adenomas to lower CRC-related deaths [13].

Stage 1 hypertension, according to the American College of Cardiology, is defined as having blood pressure at or above 130/80 mmHg [15]. Blood pressure at or above 140/90 mmHg is categorized as stage 2 hypertension [15]. The categorization of blood pressure and measurement is shown in Table 3.

**Table 3:** Blood pressure category and measurement. Source: Reference [15].

Blood pressure category	Systolic blood pressure (mmHg)	Diastolic blood pressure (mmHg)
Normal blood pressure	<120	and <80
Elevated blood pressure	120–129	and <80
Hypertension, stage 1	130–139	or 80–89
Hypertension, stage 2	≥140	or ≥90

In the United States, high blood pressure was attributed to 685,875 deaths in 2022; 48.1%, or 119.9 million adults, have stage 1 hypertension or use medication to control their blood pressure [16]. The prevalence of high blood pressure is higher in men (50%) compared to women (44%); African American adults account for 56% of the population with high blood pressure compared to 48% of White adults, 46% of Asian adults, and 39% of Hispanic adults [16]. Although African American adults have the highest rate of diagnosed high blood pressure, they represent only 25% of adults who control their blood pressure with medication; White adults account for 32%, Asian adults for 19%, and Hispanic adults for 25% [16]. Various studies have explored reasons for the higher rate of hypertension in African American adults compared to White adults. One reason cited is a genetic variation in the MYH9 region on chromosome 22, with focal segmental glomerulosclerosis found in 74% of African American adults compared to 4% of White adults [17]. Differences in dietary habits, socioeconomic status, health behaviors, and environment have also been associated with the prevalence of hypertension in African American adults compared to other races [17]. African American adults generally have reduced access to quality healthcare. The screening rate among individuals without consistent healthcare access, follow-up care, and hypertension treatment has been identified as a barrier to effective hypertensive care [18].

Study limitation

This study used EMR data from a tertiary healthcare center in Central Illinois, which may not apply to populations outside this area. The small CRC positive subgroup in the data, unmeasured cofounders, a relatively small number of racial subgroups in the capture area compared to the general United States population may make generalization of this study to the populations outside the capture area not applicable. A strength of this study is its large dataset, spanning 10 years and including diverse participants.

## Conclusions

Measuring blood pressure among African American adults could help identify individuals at a higher risk for CRC. This study validated the statistical association between CRC screening and hypertension. The data showed that African American adults had the highest percentage of hypertension and the highest rate of positive CRC tests in Illinois. This suggests that African American adults with hypertension in Illinois are at a higher risk of being diagnosed with CRC compared to other races. To reduce hypertension in this population, there should be improvements in their socioeconomic conditions. Additionally, targeted community-based screening programs for hypertensive African Americans, coupled with culturally tailored education on CRC prevention, may reduce disparities in early detection and outcomes.
